# Supplementary material for: Overcoming efficiency and stability limits in water-processing nanoparticular organic photovoltaics by minimizing microstructure defects
Source: Nat Commun. 2018 Dec 17;9:5335. doi: 10.1038/s41467-018-07807-5 (PMC6297219; doi:10.1038/s41467-018-07807-5)
Supplement: Supplementary file 1 — Supplementary Information [file 41467_2018_7807_MOESM1_ESM.docx]

**Overcoming efficiency and stability limits in water-processing nanoparticular organic photovoltaics by minimizing microstructure defects**

Xie et al.

Supplementary Figure 1. Efficiency evolution of NP dispersion-processed organic solar cells and the relevant efficiencies of solution-processed solar cells with the same active layers.

Supplementary Figure 2. SEM (scale bar: 100 nm) of surfactant-free P3HT:IDTBR NPs processed from **a**, 2-butanol and **b**, 3-hexanol. Both samples are processed by spin-coating 2.5 mg/mL dispersion on Si slides. **c**, PL of films processed by those 2 alcohols and film from chloroform solution. Excited at 500 nm. All 3 films were processed by spin-coating 10 mg/mL solution on glass slides and annealing at 150 ºC for 5 min.

Supplementary Figure 3. Chemical structure of surfactant used for NP synthesis.

Supplementary Figure 4. **F127 calibration.** Absorption and calibration curve used to determine free F127 concentration during centrifugal washing. Pluronic F127 and cobalt thiocyanate formed a dark blue complex (absorbance at 623 nm).

Supplementary Figure 5. Fourier-transform infrared spectra of P3HT:IDTBR NPs with **a**,**b**, SDS and **c**,**d**, F127 as surfactant before and after washing process.

Supplementary Figure 6. Normalized NEXAFS spectra of P3HT, IDTBR ,P3HT:IDTBR cs-NPs and P3HT:IDTBR (1:1) films.

Supplementary Figure 7. **Thermal annealing of cs-NP film.** AFM (5 × 5 μm) of as cast (left) and annealed (150 °C, 10 min) (right) films processed by P3HT:o-IDTBR cs-NPs after 5 times of centrifugal washes.

Supplementary Figure 8. Optical microscope images of as cast P3HT:IDTBR films processed from cs-NP dispersion (a) without, after (b) 1 time, (c) 2 times, (d) 3 times (e) 4 times and (f) 5 times centrifugal washes.

Supplementary Figure 9. **2D 2D-GIWAXS images** of P3HT:o-IDTBR-based as cast (**a**,**b**,**c**) and annealed (**d**,**e**,**f**) films from THF solution (**a**,**d**) SDS-stabilized dispersion (**b**,**e**) and F127-stabilized dispersion (**c**,**f**).

Supplementary Figure 10. GIWAXS profiles of as cast P3HT:IDTBR films from cs-NPs after 2 times and 5 times centrifugal washes, respectively, collected from **a**, out-of-plane cuts and **b**, in-plane cuts. As shown in Fig. S8, the NP films with less than once washing step are not homogenous, which are not appropriate for GIWAXS measurement. The film with 2 times washes contains 4% of F127. The relatively low (100), (200) and (300) peaks form out-of-plane cuts indicates that the residual surfactant would deteriorate the crystallinity of NP film.

Supplementary Figure 11. Transmission WAXS and SAXS profiles of SDS stabilized P3HT:IDTBR NP dispersion as well as cs-NP dispersion after 2 times and 5 times centrifugal washes.

Supplementary Figure 12. **a**, AFM height image of isolated P3HT:IDTBR NPs spin-coated on silicon substrate and **b**, the extracted height profiles of the numbered 9 particles.

Supplementary Figure 13. **2D-GIWAXS images of** pure **a**, as cast P3HT, **b**, as cast o-IDTBR, **c**, annealed P3HT and **d**, annealed o-IDTBR as well as their corresponding profiles from **e**, out-of-plane and **f**, in-plane cuts.

Supplementary Figure 14. Schematic overview of the structure and crystallinity of P3HT:IDTBR NP with and without surfactant during film deposition. The NPs are smeared out along the surface and deformed into a film during spin-coating. The residual surfactant on the NP surface hampers the recrystallization of polymer and NFA during NP deformation process. For the NP after surfactant-stripping, NPs prefer to merge with each other and lead to a film with high crystallinity.

Supplementary Figure 15. **Thickness dependency of P3HT:IDTBR cs-NP solar cells.** **a**, *J-V* characteristics and **b**, PCE as a function of active layer thickness of cs-NP devices.

Supplementary Figure 16. **Residual F127 in cs-NP-based organic solar cells.** **a**, Light and **b**, dark J-V characteristics and of P3HT:o-IDTBR solar cells processed by water dispersion with cs-NPs after 1, 2, 3 and 5 times of centrifugal washes.

Supplementary Figure 17. IQE of devices processed by a, water (SDS) and b, water (F127) under different bias.

Supplementary Figure 18. **PL spectra** of films after annealing at 150 °C for 10 min : **a**, experimentally measured PL of P3HT, IDTBR and blend films excited at 500 nm; **b**, measured and fitted PL of blend film processed by 3 inks.

Supplementary Figure 19. IQE of P3HT:IDTBR solar cells processed by solution and dispersion.

Supplementary Figure 20. Charge collection efficiency (*η*_cc_) of P3HT:IDTBR solar cells processed by solution and dispersion.

Supplementary Figure 21. **Photo-CELIV measurements on the P3HT:IDTBR solar cells** processed by **a**, THF, **b**, water (SDS) and **c**, water (F127) under different delay times between the light pulse and the extraction voltage ramp. **d**, Calculated extracted charge density as function of t_d_ + t_max_.

Supplementary Figure 22. **a**, *V_OC_* and **b**, *J_SC_* as function of light intensity for the P3HT:IDTBR solar cells processed by various solvents.

Supplementary Figure 23. Absolute values of **a**, Jsc, **b**, Voc, **c**, FF, and **d**, PCE of P3HT:IDTBR solar cells processed by solution and dispersion in the course of 2000 h of light exposure in N_2_.

Supplementary Figure 24. **cs-NPs with PBQ-QF:o-IDTBR and PBQ-QF:ITIC.** SEM of dried **a,** PBQ-QF:o-IDTBR and **b,** PBQ-QF:ITIC cs-NPs. Both samples were processed by spin-coating 2.5 mg/mL dispersion on Si slides. Size distribution of **c,** PBQ-QF:o-IDTBR and **d,** PBQ-QF:ITIC cs-NPs in water. F127 retention as a function of centrifugal washes at ºC in **e,** PBQ-QF:o-IDTBR and **f,** PBQ-QF:ITIC dispersion.

Supplementary Figure 25. **2D-GIWAXS images of a**, SDS-stabilized and **b**, F27- stabilized PCE10:IDTBR NP film; **c**, SDS-stabilized and **d**, F27- stabilized PBQ-QF:IDTBR NP film; **e**, SDS-stabilized and **f**, F27- stabilized PBQ-QF:ITIC NP film. All the films were annealed at 120 ºC for 10 min.

Supplementary Figure 26. **The GIWAXS profiles** of **a**,**b**, PCE10:IDTBR **c**,**d**, PBQ-QF:IDTBR and **e**,**f**, PBQ-QF:ITIC NP film collected from **a**,**c**,**d**, out-of-plane cuts and **b**,**d**,**f**, in-plane cuts. All the films were annealed at 120 ºC for 10 min.

Supplementary Figure 27. **Azimuthal integration** of **a**, P3HT:IDTBR, **b**, PCE10:IDTBR, **c**, PBQ-QF:IDTBR and **d**, PBQ-QF:ITIC NP films. All the films are annealed before GIWAXS measurement.

Supplementary Figure 28. Normalized PCE of **a**, PCE10:IDTBR, **c**, PBQ-QF:IDTBR and **d**, PBQ-QF:ITIC cs-NP based device in the course of 2000 h of light exposure in N_2_.

Supplementary Figure 29. **Light spectrum of metal halide lamp** with and without filter for solar cell stability test.

Supplementary Table 1. Reported PCEs for water/alcohol NP dispersion processed organic solar cells and their relevant PCEs of solar cells with the same active layers processed by toxic solvents.

| Year | Materials | Good solvent | Bad solvent | Surfactant | Best PCE  [%] | Reference | PCE from toxic processing [%] | Solvent | Reference |
| --- | --- | --- | --- | --- | --- | --- | --- | --- | --- |
| 2003 | F8BT:PFB | CF^a^ | Water | SDS | 0.004 | 1 | 0.2 | CF | 1 |
| 2011 | PSBTBT:PCBM | CF | Water | SDS | 0.55 | 2 | 5.1 | CB^b^ | 3 |
| 2012 | P3HT:PCBM | CF | Water | SDS | 0.29 | 4 | 3.13 | CB | 4 |
| 2012 | F8BT:PFB | CF | Water | SDS | 0.39 | 5 | 0.2 | CF | 1 |
| 2013 | P3HT:PCBM | CF | Water | SDS | 1.31 | 6 | 3.13 | CB | 4 |
| 2013 | P3HT:ICBA | CF | Water | SDS | 2.50 | 7 | 5.44 | DCB^c^ | 8 |
| 2014 | P3HT:PCBM | CF | Ethanol | - | 1.09 | 9 | 3.13 | CB | 4 |
| 2014 | PDPPTNT:PC_71_BM | CF | Water | SDS | 1.99 | 10 | 3.6 | CF+DCB | 11 |
| 2014 | P3HT:PCBM | CF | Water | SDS | 2.15 | 12 | 3.13 | CB | 4 |
| 2014 | P3HT:ICBA | CF | Ethanol | - | 3.5 | 13 | 5.44 | DCB | 8 |
| 2015 | P3HT:PCBM | CF | Water | SDS | 1.16 | 11 | 3.13 | CB | 4 |
| 2015 | P(TBT-DPP):ICBA | CF | Water | SDS | 2.63 | 15 | 4.75 | CF+DCB | 16 |
| 2016 | TQ1: PC_71_BM | CF | Water | SDS | 2.54 | 17 | 6.0 | DCB | 18 |
| 2016 | P3HT:ICBA | CF | Ethanol | - | 4.3 | 19 | 5.44 | DCB | 8 |
| 2016 | PBDTTPD:PC_71_BM | CB | Water | SDS | 3.8 | 20 | 7.9 | CB+CN^d^ | 21 |
| 2017 | PCDTBT: PC_71_BM | CB | Water | SDS | 1.9 | 22 | 6.33 | CB+DCB | 23 |
| 2017 | PDPP5T: PC_71_BM | CF | Water | SDS | 2.36 | 24 | 5.74 | CF+DCB | 25 |
| 2018 | PCDTBT:PC_71_BM | THF^e^ | Water | - | 0.33 | 26 | 6.33 | CB+DCB | 23 |
| 2018 | PDPP5T-2: PC_71_BM | CF | Water | SDS | 3.38 | 25 | 5.74 | CF+DCB | 25 |
| 2018 | P3HT:ICBA | CF | Ethanol | - | 4.52 | 27 | 5.44 | DCB | 8 |
| 2018 | PTNT:PC_71_BM | xylene | Water | SDS | 1.65 | 28 | 4.6 | DCB | 29 |
| 2018 | P3HT:o-IDTBR | THF | Water | F127 | 5.23 | This work | 6.3 | DCB | 30 |
| 2018 | PCE10:o-IDTBR | THF | Water | F127 | 5.19 | This work | 9.57 | DCB | 31 |
| 2018 | PBQ-QF:o-IDTBR | THF | Water | F127 | 6.52 | This work | - | - | - |
| 2018 | PBQ-QF:ITIC | THF | Water | F127 | 7.50 | This work | 8.90 | THF+IPA^f^ | 32 |

^a^ Chloroform. ^b^ Chlorobenzene. ^c^ ortho-Dichlorobenzene. ^d^ 1-chloronaphthalene ^e^ Tetrahydrofuran. ^f^ Isopropanol

Supplementary Table 2. Diameter of nanoparticles synthesized through nanoprecipitation method from chloroform/alcohols system. Different conjugated polymers were involved.

|  | Ethanol | 2-Propanol | 2-Butanol | 3-Hexanol | cis-3-Hexen-1-ol |
| --- | --- | --- | --- | --- | --- |
| P3HT:ICBA^a^ | 104 nm | 95 nm | 90 nm | 83 nm | 79 nm |
| P3HT:IDTBR | Aggregates | Aggregates | 110 nm | 99 nm | Aggregates |
| PDCBT | Aggregates | Aggregates | Aggregates | Aggregates | Aggregates |
| PCDTBT | Aggregates | Aggregates | Aggregates | Aggregates | Aggregates |
| PDPP5T-2 | Aggregates | Aggregates | Aggregates | Aggregates | Aggregates |
| PDPP-TT-T | Aggregates | Aggregates | Aggregates | Aggregates | Aggregates |
| PTB7 | Aggregates | Aggregates | Aggregates | Aggregates | 170 nm |
| PCE10 | Aggregates | Aggregates | Aggregates | Aggregates | Aggregates |
| PCE11 | Aggregates | Aggregates | Aggregates | Aggregates | Aggregates |

^a^ Data is obtained from Supplementary Reference 20.

Supplementary Table 3. Zeta potential (mV) of P3HT:IDTBR NP dispersions synthesized by various surfactants during centrifugal washes.

|  | Surfactant^a^ | | | | | |
| --- | --- | --- | --- | --- | --- | --- |
| Wash number | F127 (0 °C) | F127 (25 °C) | SDS (0 °C) | SDS (25 °C) | DTAB | Brij |
| 0 | -40.5 | -40.5 | -69 | -69 | 50.8 | -39.5 |
| 1 | -27.7 | -34.7 | -62 | -58.3 | 40.9 | -38.3 |
| 2 | -12.1 | -30.7 | -58.3 | -52.4 | 36 | -31.5 |
| 3 | -11 | -28 | -55.5 | -48.4 | 34 | -30 |
| 4 | -11.1 | -22.8 | -51.5 | -47.3 | 31.5 | -26.2 |
| 5 | -10.1 | -20.8 | -49 | -43.8 | 30.1 | -25.9 |
| 6 | -10.8 | -19.2 | -43.9 | -38.2 | 24.8 | -21.5 |
| 7 | -11 | -20 | -43.9 | -34.3 | 26 | -18 |
| 8 | -10 | -19.6 | -37.7 | -34.9 | 23.3 | -17.6 |
| 9 | -10.3 | -20.1 | -33.8 | -35.9 | 20.9 | -17.6 |
| 10 | -10.3 | -20.2 | -35.1 | -34.6 | 21.8 | -17 |

^a^ Not including PSMA and PMMA. We found that centrifugal filtration could not remove those 2 surfactants from water dispersion. PSMA has low solubility in water and PMMA hardly goes through the centrifugal filter. The zeta potential keeps their initial value even after several washes.

Supplementary Table 4. IQE, exciton harvesting efficiencies, and charge collection efficiencies of devices.

|  | IQE^a^  [%] | *η*_eh,D_  [%] | *η*_eh,A_  [%] | *η*_cc_^a^  [%] |
| --- | --- | --- | --- | --- |
| THF | 45.2 | 74.9 | 68.8 | 65.5 |
| Water (SDS) | 42.7 | 70.6 | 71.7 | 59.6 |
| Water (F127) | 53.8 | 76.3 | 72.4 | 74.2 |

^a^ IQE or *η*_cc_ value at a wavelength of 730 nm.

Supplementary Table 5. EQE calibrated *J_SC_*, *R*_s_ and *R_sh_* of solar cells processed by various solutions and dispersions. All the integrated EQE spectra with AM1.5G solar spectrum match well with the *J_SC_* measured under a solar simulator with a margin of less than 5%.

| Active layer | Solvent | *J*_SC_ *J-V*  [mA cm^-2^] | *J*_SC_ EQE  [mA cm^-2^] | *R*s  [Ohm cm^2^] | *R*sh  [kOhm cm^2^] |
| --- | --- | --- | --- | --- | --- |
| P3HT:IDTBR | THF | 8.72 | 8.50 | 17.096 | 1.63E4 |
| P3HT:IDTBR | Water (SDS) | 7.67 | 7.38 | 104.756 | 2.12E4 |
| P3HT:IDTBR | Water (F127) | 10.68 | 10.42 | 3.864 | 5.21E4 |
| PCE10:IDTBR | Water (F127) | 11.81 | 11.72 | 10.782 | 5.10 |
| PBQ-QF:IDTBR | Water (F127) | 13.44 | 13.44 | 2.448 | 1.78 |
| PBQ-QF:ITIC | Water (F127) | 15.36 | 15.17 | 1.425 | 1.27 |

Supplementary Note 1. **Synthesis of alcoholic NPs:** The synthesis follows the nanoprecipitation approach in Supplementary Reference 10. The precursor solution was prepared by dissolving polymers in chloroform and stirring overnight. Then, the solution (5 mg/mL) was add dropwisely to the alcohols (1:4, v:v) at 60 °C under vigorous stirring. Right after precipitation, the good solvent was removed by heating the dispersion to 65 °C without stirring. The size of NPs were summarized in Supplementary Table 2.

Supplementary Note 2. **Quantification of surfactant by FT-IR spectra:** As shown in Supplementary Figure 5, The FTIR spectra was calibrated by the peaks around 1700-1400 cm-1, which stand for the stretching vibration of C-C in the thiophenic ring in polymer PDPP5T-2. The SDS and F127 was quantitively evaluated by the peak area of S=O stretching at 1185 cm^-1^ and C-O-C stretching at 1117 cm^-1^ . The peak area is proportional to the product of peak width and peak height, which can be obtained from those spectrums.

Supplementary Note 3. **NEXAFS spectra:** As shown in Supplementary Figure 6, the spectra of cs-NPs film is close to that from a film with solution-processed with P3HT:IDTBR exactly 1:1. It is evidence that there is almost no material loss during the NP synthesis.

Supplementary Note 4. **Transmission X-ray scattering:** As show in Supplementary Figure 11, three dispersions, SDS-stabilized NP, cs-NP after 2 time centrifugal washes and cs-NP after 5 times washes were measured by transmission WAXS and SAXS. The transmission WAXS and SAXS In the transmission WAXS measurement of the aqueous nanoparticle dispersions an intensive peak at 3.6 nm^-1^ is observed in all three dispersions. The SAXS patterns of the three samples studied were found to be very similar. Fitting the experimental data by applying a model of isolated compact and homogeneous spheres with a Gaussian shaped size distribution the data can nicely be reproduced. The diameters of NPs in the three samples are similar. These characterizations of NP dispersion are not able to reflect the exact crystalline of NPs. The polymer and NFA in NPs maybe randomly orienting when they are dispersed in water.

Supplementary Note 5. **NP deformation:** The diluted P3HT:IDTBR cs-NP dispersion (1 mg/mL) was spin-coated (1000 rpm) on Si substrate. As shown in Supplementary Figure 12, the selected 9 particles all exhibit a higher value of width than that of height. This observation suggests a deformation occurs during spin-coating those soft polymer:NFA colloids. This deformation would drive a crystallization of NP films, which is corresponded to the observation in Supplementary Reference 14.

Supplementary Note 6. **Charge collection efficiency calculation:** As IQE is the probability of an absorbed photon resulting in an electron being collected at the electrode, there are several processes that could prove to be the main reason behind the comparatively lower IQE in devices. After the photon absorption creates an exciton, the exciton must first diffuse to the interface (exciton diffusion) where it undergoes a charge transfer process resulting in the electron on the acceptor and hole on the donor (charge transfer). The combined processes of exciton diffusion and charge transfer are referred to as exciton harvesting (EH), which occurs with an efficiency *η_eh_*. From the charge transfer (CT) state, the electron and hole must be separated into free charges and collected at the metal electrodes. The combined processes of CT state separation and free carrier collection are referred to as charge collection (CC), which occurs with efficiency *η_cc_*.

The exciton harvesting efficiency, *η_eh_*, can be estimated by examining the photoluminescence (PL) signal of a blended donor-acceptor film compared to neat films of the pure donor and acceptor. Blended films with efficient exciton harvesting exhibit a PL signal that is nearly completely quenched compared to the pure material. The PL spectra from the blended film can be modeled as a linear combination of the PL spectra of the two pure films, as given by Supplementary Equation 1 and 2:^33^

$$\mathrm{PL}_{blend}\left( \lambda\right)=\left( 1-\eta_{eh, D} \right)f_{D}\mathrm{PL}_{D}\left( \lambda\right)+\left( 1-\eta_{eh,A} \right)f_{A}\mathrm{PL}_{A}\left( \lambda\right) (1)$$

$$f_{D/A}=\frac{x_{D/A}k_{D/A}\left( \lambda\right)}{x_{D}k_{D}\left( \lambda\right)+x_{A}k_{A}\left( \lambda\right)} (2)$$

Where *η*_eh_ is the exciton harvesting efficiency, f is the fraction of photons absorbed, PL is the photo-luminescence spectrum, x is the volume fraction and k is the extinction coefficient. k can be obtained from absorbance. All variables can refer to either the donor (D) or acceptor (A) phase. Fitting Supplementary Equation 1 to the data in Supplementary Figure 18, we calculate an exciton harvesting efficiency in the P3HT:o-IDTBR system shown in Supplementary Table 4.

Once *η_eh_* in the donor and acceptor phases is known, the IQE spectrum can be used to calculate the charge collection efficiency, *η_cc_* , according to Supplementary Equation 3 and 4 :

$$\mathrm{IQE}\left( \lambda\right)=\eta_{eh}\eta_{cc}=\left( \eta_{eh,D}f_{D}\left( \lambda\right)+\eta_{eh,A}f_{A}\left( \lambda\right) \right)\eta_{cc} (3)$$

$$\eta_{cc}=\frac{\mathrm{IQE}\left( \lambda\right)}{\left( \eta_{eh,D}f_{D}\left( \lambda\right)+\eta_{eh,A}f_{A}\left( \lambda\right) \right)} (4)$$

In this model, two assumptions should be satisfied:

1. The crystallinity of donor and acceptor in all the films are identical.
2. The quantum efficiency of PL is identical in any wavelength.

Due to the uncertain factors shown above, there would be considerable discrepancy between the calculated values and exact values. The PL spectra of neat P3HT, neat o-IDTBR and the three blend films can be found in Supplementary Figure 18. Fitting Supplementary equation 1 to the data, we calculated the exciton efficiency in all blend systems for both, the donor (*η*_eh.D_, P3HT) and the acceptor (*η*_eh,A_, IDTBR) (Supplementary Table 4). Fairly similar exciton harvesting efficiencies was observed for all three systems. Moreover, the fitted charge collection efficiency as a function of wavelength calculated from the IQE (Supplementary Figure 19) and Supplementary Equation 2-4 is shown in Supplementary Figure 20.

**Supplementary References**

1. Kietzke, T. *et al.* Novel approaches to polymer blends based on polymer nanoparticles. *Nat. Mater.* **2,** 408–12 (2003).

2. T.R. Andersen, T.T. Larsen-Olsen, B. Andreasen, A.P. L. Böttiger, J.E. Carl, M. Helgesen, E. Bundgaard, K. Norrman, J.W. Andreasen, M. Jorgensen, F. C. K. Aqueous Processing of Low-Band-Gap Polymer Solar Cells Using Roll-to-Roll Methods. *ACS Nano* **5,** 4188–4196 (2011).

3. Hou, J., Chen, H.-Y., Zhang, S., Li, G. & Yang, Y. Synthesis, Characterization, and Photovoltaic Properties of a Low Band Gap Polymer Based on Silole-Containing Polythiophenes and 2,1,3-Benzothiadiazole. *J. Am. Chem. Soc.* **130,** 16144–16145 (2008).

4. Larsen-Olsen, T. T. *et al.* Simultaneous multilayer formation of the polymer solar cell stack using roll-to-roll double slot-die coating from water. *Sol. Energy Mater. Sol. Cells* **97,** 22–27 (2012).

5. Stapleton, A. *et al.* A multilayered approach to polyfluorene water-based organic photovoltaics. *Sol. Energy Mater. Sol. Cells* **102,** 114–124 (2012).

6. Ulum, S. *et al.* Determining the structural motif of P3HT:PCBM nanoparticulate organic photovoltaic devices. *Sol. Energy Mater. Sol. Cells* **110,** 43–48 (2013).

7. Ulum, S. *et al.* The role of miscibility in polymer: Fullerene nanoparticulate organic photovoltaic devices. *Nano Energy* **2,** 897–905 (2013).

8. He, Y., Chen, H.-Y., Hou, J. & Li, Y. Indene−C 60Bisadduct: A New Acceptor for High-Performance Polymer Solar Cells. *J. Am. Chem. Soc.* **132,** 1377–1382 (2010).

9. Darwis, D. *et al.* Surfactant-free nanoparticulate organic photovoltaics. *Sol. Energy Mater. Sol. Cells* **121,** 99–107 (2014).

10. Vaughan, B. *et al.* Water-based nanoparticulate solar cells using a diketopyrrolopyrrole donor polymer. *Phys. Chem. Chem. Phys.* **16,** 2647–53 (2014).

11. Williams, E. L. *et al.* Nanoscale phase domain structure and associated device performance of organic solar cells based on a diketopyrrolopyrrole polymer. *RSC Adv.* **3,** 20113–20124 (2013).

12. Gehan, T. S. *et al.* Multiscale active layer morphologies for organic photovoltaics through self-assembly of nanospheres. *Nano Lett.* **14,** 5238–5243 (2014).

13. Gärtner, S. *et al.* Eco-friendly fabrication of 4% efficient organic solar cells from surfactant-free P3HT:ICBA nanoparticle dispersions. *Adv. Mater.* **26,** 6653–6657 (2014).

14. Pedersen, E. B. L. *et al.* Structure and crystallinity of water dispersible photoactive nanoparticles for organic solar cells. *J. Mater. Chem. A* **3,** 17022–17031 (2015).

15. Yamamoto, N. A. D. *et al.* Charge transport model for photovoltaic devices based on printed polymer: Fullerene nanoparticles. *Sol. Energy Mater. Sol. Cells* **141,** 171–177 (2015).

16. Pierre, A., Lu, S., Howard, I. A., Facchetti, A. & Arias, A. C. Empirically based device modeling of bulk heterojunction organic photovoltaics. *J. Appl. Phys.* **113,** (2013).

17. Holmes, N. P. *et al.* Nano-pathways: Bridging the divide between water-processable nanoparticulate and bulk heterojunction organic photovoltaics. *Nano Energy* **19,** 495–510 (2016).

18. Wang, E. *et al.* An easily synthesized blue polymer for high-performance polymer solar cells. *Adv. Mater.* **22,** 5240–5244 (2010).

19. Sankaran, S. *et al.* Fabrication of polymer solar cells from organic nanoparticle dispersions by doctor blading or ink-jet printing. *Org. Electron.* **28,** 118–122 (2016).

20. D’Olieslaeger, L. *et al.* Eco-friendly fabrication of PBDTTPD:PC71BM solar cells reaching a PCE of 3.8% using water-based nanoparticle dispersions. *Org. Electron.* **42,** 42–46 (2016).

21. Pirotte, G. *et al.* Continuous Flow Polymer Synthesis toward Reproducible Large-Scale Production for Efficient Bulk Heterojunction Organic Solar Cells. *ChemSusChem* **8,** 3228–3233 (2015).

22. D’Olieslaeger, L. *et al.* Tuning of PCDTBT:PC71BM blend nanoparticles for eco-friendly processing of polymer solar cells. *Sol. Energy Mater. Sol. Cells* **159,** 179–188 (2017).

23. Sun, Y., Seo, J. H., Takacs, C. J., Seifter, J. & Heeger, A. J. Inverted polymer solar cells integrated with a low-temperature-annealed sol-gel-derived ZnO film as an electron transport layer. *Adv. Mater.* **23,** 1679–1683 (2011).

24. Colberts, F. J. M., Wienk, M. M. & Janssen, R. A. J. Aqueous Nanoparticle Polymer Solar Cells: the Effects of Surfactant Concentration and Processing on Device Performance. *ACS Appl. Mater. Interfaces* **9,** 13380–13389 (2017).

25. Xie, C. *et al.* Overcoming Microstructural Limitations in Water Processed Organic Solar Cells by Engineering Customized Nanoparticulate Inks. *Adv. Energy Mater.* **8,** 1702857 (2018).

26. Prunet, G. *et al.* Aqueous PCDTBT:PC _71_ BM Photovoltaic Inks Made by Nanoprecipitation. *Macromol. Rapid Commun.* **39,** 1700504 (2018).

27. Xie, C. *et al.* Robot-Based High-Throughput Engineering of Alcoholic Polymer: Fullerene Nanoparticle Inks for an Eco-Friendly Processing of Organic Solar Cells. *ACS Appl. Mater. Interfaces* **10,** 23225−23234 (2018).

28. Pan, X. *et al.* Environmentally friendly preparation of nanoparticles for organic photovoltaics. *Org. Electron.* **59,** 432–440 (2018).

29. Kroon, R. *et al.* A new tetracyclic lactam building block for thick, broad-bandgap photovoltaics. *J. Am. Chem. Soc.* **136,** 11578–11581 (2014).

30. Holliday, S. *et al.* High-efficiency and air-stable P3HT-based polymer solar cells with a new non-fullerene acceptor. *Nat. Commun.* **7,** 11585 (2016).

31. Hoefler, S. F. *et al.* The effect of polymer molecular weight on the performance of PTB7-Th:O-IDTBR non-fullerene organic solar cells. *J. Mater. Chem. A* **6,** 9506–9516 (2018).

32. Zheng, Z. *et al.* Efficient Charge Transfer and Fine-Tuned Energy Level Alignment in a THF-Processed Fullerene-Free Organic Solar Cell with 11.3% Efficiency. *Adv. Mater.* **29,** 3–8 (2017).

33. Bloking, J. T. *et al.* Comparing the device physics and morphology of polymer solar cells employing fullerenes and non-fullerene acceptors. *Adv. Energy Mater.* **4,** 1301426 (2014).
