# Supplementary material for: Overcoming efficiency and stability limits in water-processing nanoparticular organic photovoltaics by minimizing microstructure defects
Source: Nat Commun. 2018 Dec 17;9:5335. doi: 10.1038/s41467-018-07807-5 (PMC6297219; doi:10.1038/s41467-018-07807-5)
Supplement: Supplementary file 3 — Solar cells reporting summary [file 41467_2018_7807_MOESM3_ESM.pdf]

nature research

Corresponding author(s): Chen Xie, Ning Li and Christoph J. Brabec

Solar Cells Reporting Summary

Nature Research wishes to improve the reproducibility of the work that we publish. This form is intended for publication with all accepted papers reporting the characterization of photovoltaic devices and provides structure for consistency and transparency in reporting. Some list items might not apply to an individual manuscript, but all fields must be completed for clarity.

For further information on Nature Research policies, including our data availability policy, see Authors & Referees.

Experimental design

Please check: are the following details reported in the manuscript?

1. Dimensions

Area of the tested solar cells
- [x] Yes — Described in Methods, section "Device".
- [ ] No — *Explain why this information is not reported/not relevant.*

Method used to determine the device area
- [x] Yes — Described in Methods, section "Device".
- [ ] No — *Explain why this information is not reported/not relevant.*

2. Current-voltage characterization

Current density-voltage (J-V) plots in both forward and backward direction
- [ ] Yes — *State where this information can be found in the text.*
- [x] No — Solar cells were evaluated in forward direction

Voltage scan conditions
For instance: scan direction, speed, dwell times
- [x] Yes — Described in Methods, section "Device".
- [ ] No — *Explain why this information is not reported/not relevant.*

Test environment
For instance: characterization temperature, in air or in glove box
- [x] Yes — Described in Methods, section "Device".
- [ ] No — *Explain why this information is not reported/not relevant.*

Protocol for preconditioning of the device before its characterization
- [ ] Yes — *State where this information can be found in the text.*
- [x] No — No preconditioning of the device before its characterization.

Stability of the J-V characteristic
Verified with time evolution of the maximum power point or with the photocurrent at maximum power point; see ref. 7 for details.
- [x] Yes — Provided in Fig. 4g.
- [ ] No — *Explain why this information is not reported/not relevant.*

3. Hysteresis or any other unusual behaviour

Description of the unusual behaviour observed during the characterization
- [ ] Yes — *State where this information can be found in the text.*
- [x] No — No unusual behavior.

Related experimental data
- [ ] Yes — *State where this information can be found in the text.*
- [x] No — No unusual behavior.

4. Efficiency

External quantum efficiency (EQE) or incident photons to current efficiency (IPCE)
- [x] Yes — Provided in Fig. 4 and Fig. 5
- [ ] No — *Explain why this information is not reported/not relevant.*

A comparison between the integrated response under the standard reference spectrum and the response measure under the simulator
- [x] Yes — Provided in Supplementary Table 5.
- [ ] No — *Explain why this information is not reported/not relevant.*

For tandem solar cells, the bias illumination and bias voltage used for each subcell
- [ ] Yes — *State where this information can be found in the text.*
- [x] No — No tandem solar cells are involved in this manuscript.

5. Calibration

Light source and reference cell or sensor used for the characterization
- [x] Yes — Described in Methods, section "Device".
- [ ] No — *Explain why this information is not reported/not relevant.*

Confirmation that the reference cell was calibrated and certified

- [x] Yes — Described in Methods, section "Device".
- [ ] No — *Explain why this information is not reported/not relevant.*

Calculation of spectral mismatch between the reference cell and the devices under test

- [ ] Yes — *State where this information can be found in the text.*
- [x] No — Spectral mismatch was not calculated. The intensity of solar simulator was adjusted using a standard Si diode, as described in Methods.

6. Mask/aperture

Size of the mask/aperture used during testing

- [x] Yes — Described in Methods, section "Device".
- [ ] No — *Explain why this information is not reported/not relevant.*

Variation of the measured short-circuit current density with the mask/aperture area

- [ ] Yes — *State where this information can be found in the text.*
- [x] No — Only the 0.104 cm2 mask was used for evaluation.

7. Performance certification

Identity of the independent certification laboratory that confirmed the photovoltaic performance

- [ ] Yes — *State where this information can be found in the text.*
- [x] No — The highest efficiency value in this work is 7.5 %, which is not a high performance in today's OPV. This work focuses on the fundamental characterizations and device physics of nanoparticle solar cells.

A copy of any certificate(s)
Provide in Supplementary Information

- [ ] Yes — *State where this information can be found in the text.*
- [x] No — Not certificate.

8. Statistics

Number of solar cells tested

- [x] Yes — Described in Methods, section "Device".
- [ ] No — *Explain why this information is not reported/not relevant.*

Statistical analysis of the device performance

- [x] Yes — Provided in Table 1.
- [ ] No — *Explain why this information is not reported/not relevant.*

9. Long-term stability analysis

Type of analysis, bias conditions and environmental conditions
For instance: illumination type, temperature, atmosphere humidity, encapsulation method, preconditioning temperature

- [x] Yes — Described in Methods, section "Device".
- [ ] No — *Explain why this information is not reported/not relevant.*
